# Using Machine Learning to Collect and Facilitate Remote Access to Biomedical Databases: Development of the Biomedical Database Inventory

**DOI:** 10.2196/22976

**Published:** 2021-02-25

**Authors:** Eduardo Rosado, Miguel Garcia-Remesal, Sergio Paraiso-Medina, Alejandro Pazos, Victor Maojo

**Affiliations:** 1 Biomedical Informatics Group School of Computer Science Universidad Politecnica de Madrid Madrid Spain; 2 Grupo de Redes de Neuronas Artificiales y Sistemas Adaptativos - Imagen Médica y Diagnóstico Radiológico Department of Computer Science and Information Technologies, Faculty of Computer Science University of A Coruña A Coruña Spain

**Keywords:** biomedical databases, natural language processing, deep learning, internet, biomedical knowledge

## Abstract

**Background:**

Currently, existing biomedical literature repositories do not commonly provide users with specific means to locate and remotely access biomedical databases.

**Objective:**

To address this issue, we developed the Biomedical Database Inventory (BiDI), a repository linking to biomedical databases automatically extracted from the scientific literature. BiDI provides an index of data resources and a path to access them seamlessly.

**Methods:**

We designed an ensemble of deep learning methods to extract database mentions. To train the system, we annotated a set of 1242 articles that included mentions of database publications. Such a data set was used along with transfer learning techniques to train an ensemble of deep learning natural language processing models targeted at database publication detection.

**Results:**

The system obtained an F1 score of 0.929 on database detection, showing high precision and recall values. When applying this model to the PubMed and PubMed Central databases, we identified over 10,000 unique databases. The ensemble model also extracted the weblinks to the reported databases and discarded irrelevant links. For the extraction of weblinks, the model achieved a cross-validated F1 score of 0.908. We show two use cases: one related to “omics” and the other related to the COVID-19 pandemic.

**Conclusions:**

BiDI enables access to biomedical resources over the internet and facilitates data-driven research and other scientific initiatives. The repository is openly available online and will be regularly updated with an automatic text processing pipeline. The approach can be reused to create repositories of different types (ie, biomedical and others).

## Introduction

Since the inception of the web, the amount of information available online has dramatically increased. Such an explosion can be mainly observed in the biomedical area, where the publication of the Human Genome Project [1] led to a myriad of new primary and translational projects. The latter produced a vast amount of additional “omics” information that needed to be remotely found, accessed, collected, managed, analyzed, and used. This acceleration in data production has been observed in biology and bioinformatics in particular [2,3].

To facilitate access to such a plethora of information, thousands of different databases were created by many scientists to exchange their knowledge and data with other colleagues and institutions. The number of database publications is continuously increasing; therefore, giving visibility and access to those resources can be a complicated task. A study on the usage of databases and software in articles available at PubMed Central (PMC) [4] found that the top 5% most popular resources accounted for 47% of all citations. In comparison, 70% of all detected resources were referenced just once. This focus on a few popular resources suggests a wasted opportunity for researchers to benefit from many informatics tools designed and published to support scientific research and clinical practice.

A successful biomedical information classification initiative was the Unified Medical Language System (UMLS) [5]. Led by Lindberg, Humphreys, McCray, and staff from the National Library of Medicine (NLM), UMLS’s original mission was to facilitate computer programs in understanding and accessing biomedical literature. However, the identification of databases in scientific articles is not straightforward within UMLS. This initiative focuses on the description of scientific research and the grouping of data under big repositories for clinical and genetic data, such as the Systematized Nomenclature of Medicine (SNOMED) [6] and Online Mendelian Inheritance in Man (OMIM) [7], while neglecting smaller databases that comprise the vast majority of the available resources.

Currently, most efforts toward detecting and including new databases in repositories have relied on manual approaches. Such a strategy involving human resources cannot scale properly. Previous work in manual database compilation includes, for instance, the Database of Databases (DoD2007) [8]. The DoD2007 repository has increased over the years, having reached a total of 1082 molecular biology databases at the time of writing. The journal Nucleic Acids Research (NAR) releases a yearly update on their molecular biology database collection, with the 2020 publication [9] containing a total of 148 scientific articles either presenting a new database or reporting an update to a previously existing database. Another study [10] collected a set of 112 widely used human-related biological databases. Fairsharing (formerly Biosharing) [11] is a regularly updated, curated, and crowdsourced collection of life sciences resources. As of May 2020, it contains 1470 databases as claimed on their webpage.

**Figure 1:**
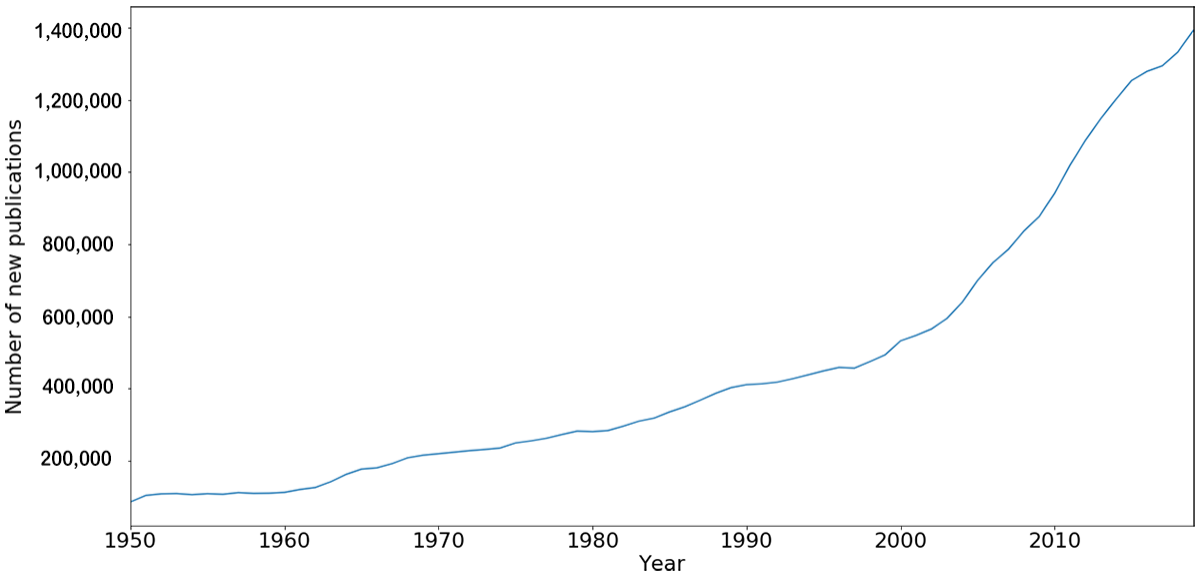
Evolution of new publications in PubMed (ie, the MEDLINE database) per year from 1950 to 2018.

The resources mentioned have been of great use for the research community. However, using manual labor for such collection efforts is a costly endeavor. Figure 1 shows the number of new publications indexed in PubMed each year, a density distribution instead of a cumulative one. As can be seen, the publication rate has been accelerating continuously, especially since the start of the new millennium. In 1950, PubMed registered 85,792 new publications; in 2019, however, 1,392,830 new publications were registered. Such numbers imply an increase of 1623% in publication rate. Since the year 2011, more than one million articles have been registered on the platform each year.

In this context, we consider automatic methods for information extraction to be a good alternative, if not a necessary one. Here we present the Biomedical Database Inventory (BiDI), a collection of databases automatically extracted from the scientific literature by applying deep learning methods for natural language processing (NLP).

## Methods

### Model Architecture

To detect databases in the literature, we have created an ensemble of NLP models adapted from different reported architectures [12-14] for masked word prediction.

**Figure 2:**
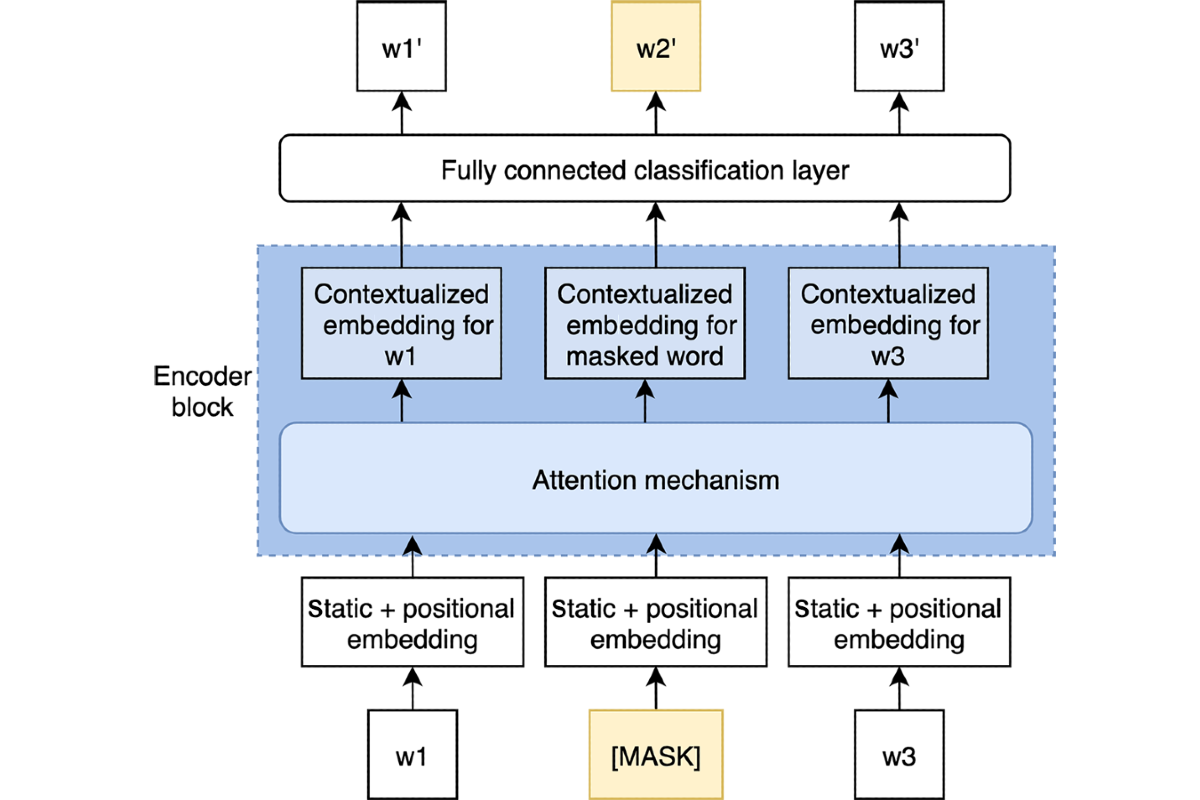
The pretraining setup for the masked word (w) prediction architecture. Only one encoder block is shown. The sample sentence only contains three words. However, in practice each sentence has a length of 512, adding padding to the right when necessary. [MASK] is a special token that is used to replace a word from the sentence.

In Figure 2, we show a simplified version of the training process for masked word prediction. Initially, we replace a word from the sentence with the special token [MASK]. Each input token is passed through a token embedding layer, thus converting each token into a vector representation. At this stage, [MASK] is just another input token and is represented by a single embedding, which is the same for all [MASK] tokens. These vectors are general word embeddings, which are randomly initialized and trained jointly with the rest of the network using gradient descent. The latter propagates through the entire network to the input token embeddings, including the [MASK] token, and, thus, they are updated just like any other model parameter.

The network also learns positional embeddings, which refer to the token sequence of input. These are represented as a combination of sine and cosine functions of different frequencies, which provide the model with information regarding the token’s situation. Finally, each token is replaced by the sum of the static token embedding and the positional embedding.

Then, we apply the attention mechanism by which a contextualized embedding is produced for each word. This embedding is a weighted sum of all input vectors multiplied by a value matrix. The weight factor would be the attention score computed for each word. Finally, each of the contextualized vectors is fed to a fully connected layer to produce a new word. The loss is calculated as the cross-entropy loss between the masked word and the model’s output in that position, discarding the predictions for all nonmasked terms. We refer to Liu et al [12] and Devlin et al [14] for specific details about the underlying architecture.

By masking 15% of every sentence repeatedly with different masking schemes each time, the model learns to predict any word in any sentence. When the training process involves millions of sentences, the result is a general-purpose language model capable of capturing complex dependencies between words. We shall remark that the particular architecture we applied has a total of 12 encoder blocks, stacked one on top of another, and a replicated attention mechanism with 12 attention heads on each encoder block. Therefore, 12 different attention schemes are applied simultaneously to focus on different input aspects.

**Figure 3:**
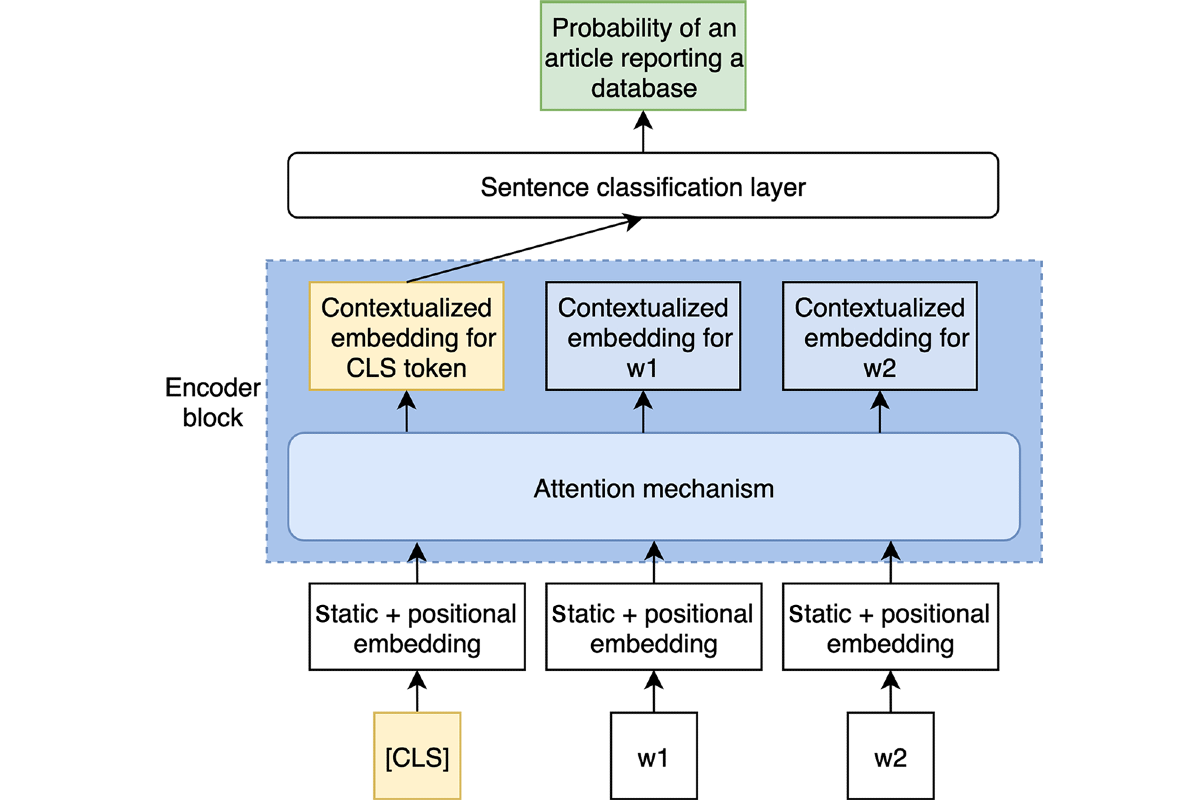
Fine-tuning the setup for our model. Only one encoder block is shown. The sample sentence only contains three words (w). However, in practice each sentence has a length of 512, adding padding to the right when necessary. [CLS]: classification token.

Our task is an instance of sentence classification. Therefore, we do not need to consider the final contextualized embeddings of each word. Instead, we can perform a forward pass on the model and extract only the first embedding, which corresponds to the special classification token [CLS]. The [CLS] token goal is to mark the start of a sentence, and it can be easily fine-tuned to represent global information about the whole input. In Figure 3, we represent our fine-tuning process.

As shown in the figure, we extract the [CLS] token and feed it to a new, fully connected classification layer to obtain a probability for the whole sentence. However, we update the parameter’s weights on the classification layer and every parameter in the encoder blocks by backpropagation. Therefore, the model transfers the relevant information regarding our task into the [CLS] embedding. Each encoder block adds information to that vector before feeding it to the classification layer.

We trained three different models under the same architecture. First, we developed a model to detect a database publication considering only the title of the article. Then we trained a second model to perform the same task with sentences extracted from the abstract. After considering the particular structure of a title sentence, we committed to this design, which usually contains vital information about the article. We aimed to train a model that focused on title sentences to extract all the information about them. Finally, we trained a model on the task of database link classification (ie, to differentiate between sentences with a link to a database homepage from sentences with a link unrelated to the database). This third model allows us to extract the right link from every article, achieving our goal of directly linking articles to data.

**Figure 4:**
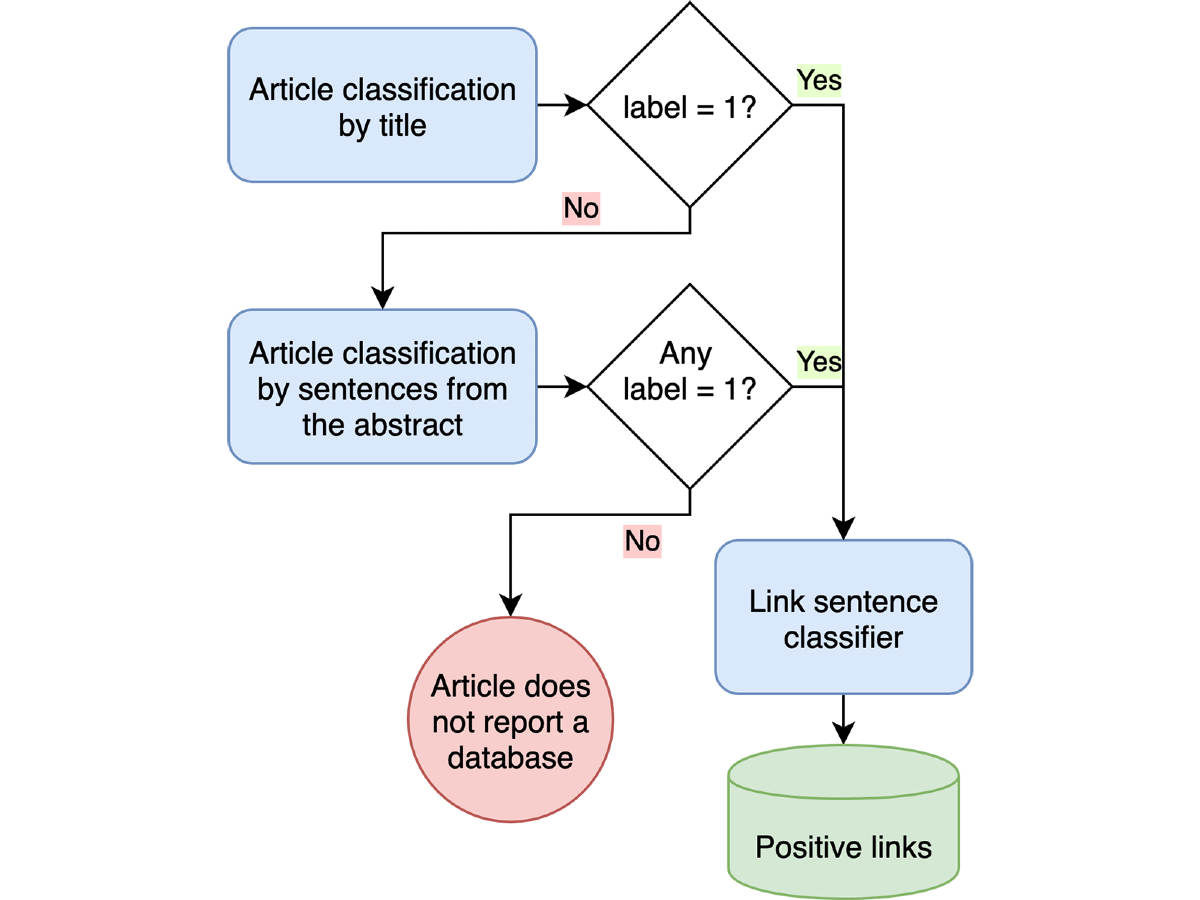
Pipeline for article classification.

In Figure 4, we represent the complete procedure, which consists of an ensemble of the three models. The first step is to classify an article by the title; only when it is classified as a negative sample do we resort to the abstract sentences for confirmation. The third model is applied to sentences containing a link only if either the first or the second model delivered a positive label for the article.

### Data Collection and Preprocessing

We performed database publication detection on two scientific article repositories: PubMed and PMC. For PubMed, we downloaded a total of 12,615,511 articles with abstracts. For PMC, we downloaded the Open Access Subset with a total of 2,710,216 articles. Aside from the number of manuscripts, the main difference between these data sources is that PMC offers full-text articles, while PubMed only provides titles and abstracts.

**Figure 5:**
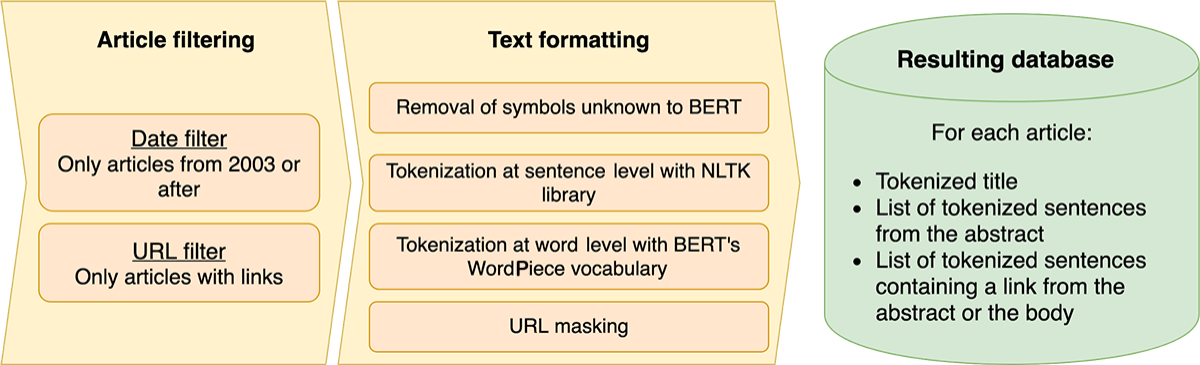
Data preprocessing steps. The subtasks inside each step were executed in order from top to bottom. BERT: Bidirectional Encoder Representations from Transformers; NLTK: Natural Language Toolkit.

Article data were subject to a series of preprocessing steps before feeding them to the model for training. The process is depicted in Figure 5.

We now describe each step in the preprocessing pipeline:

1. Article filtering. We only considered articles starting from 2003 since this was the year that marked the completion of the Human Genome Project [1], a milestone for the life sciences research community. We also removed articles not containing at least one URL, as we aimed to link the publication to the database. The URLs were extracted by applying a regular expression, using the substrings “http” and “www” as anchors.
2. Text formatting:
  - Removal of symbols. The model used a subword vocabulary of about 30,000 items. Such a vocabulary included all the letters in the English language, making it possible for the model to represent any English word, even if it was never seen by the model before, as, ultimately, it can decompose it into each letter. For languages with an alphabet different from the one used for the English language, however, the coverage was not guaranteed. We removed such foreign characters.
  - Sentence tokenization. We used the Natural Language Toolkit library for Python (Python Software Foundation) to split the whole text into smaller sentences. This library provides a rule-based system that carries out this partition process while maintaining each sentence’s semantic integrity.
  - Word tokenization. Words in the text were tokenized according to WordPiece tokens, expanding every article’s word count each time a word was split into several subwords.
  - URL masking. In our experiments, we found that URL links are hard to process for the model. We wanted the model to know that a link is present in the sentence. For this reason, we decided to mask all URLs with the keyword “link.” At the same time, we stored the original URL links to retrieve them later.

In the end, the preprocessing stage produced a database with three different fields for each article. The first one contained the title sentence, the second one consisted of all the abstract sentences, and the third one included all sentences containing a link. The third field’s sentences were extracted from the abstract for PubMed articles and from the abstract and the body for PMC articles. The preprocessing stage resulted in a reduced set of papers: 24,437 manuscripts for PubMed and 450,777 for PMC. The PubMed data set reduction was quite drastic, as it is not a common practice to include a link to the resource in the abstract. This is a typical pattern that, in our opinion, restricts the capabilities of search engines to find such information and direct access to the reported databases, since PubMed only provides the abstract.

### Building a Labeled Data Set

We compiled three annotated data sets, one for each of the models we trained. All data sets were developed from the same set of 1242 articles. We created labels for each analyzed manuscript for its title, abstract, and link sentences before considering the next one. All tags were either positive or negative, as in binary classification. The annotation was performed by two human annotators with biomedical informatics backgrounds—one of them also has formal training in the medical field. Each human annotator independently annotated the data sets. The degree of agreement regarding the annotations’ results for each category (ie, titles, abstract sentences, and link sentences) was assessed using Cohen κ. We obtained an almost perfect consensus for each of the categories.

In Table 1, we provide the count of positive and negative annotations performed. As can be seen, there was a significant data imbalance favoring the negative samples. The articles’ real distribution was actually even more skewed toward negative samples. Therefore, we believe that our data sets suitably represented the actual data distribution. Due to this imbalance, it was not easy to manually find positive articles. We actively facilitated the annotation of positive examples by discarding any negative examples we found after sampling 1000 manuscripts (ie, after the 1000^th^ sample, we only added positive examples to the data set). We continued sampling until we collected a reasonable number of positive samples so that the model could learn the syntactic and semantic patterns of the target group.

**Table 1:** Positive and negative samples, the total number of labels for each data field, and annotator agreement evaluation per category. In all cases, the unit for the count is a sentence.

Category	Positive samples, n (%)	Negative samples, n (%)	Total, N (%)	Annotator agreement, Cohen κ
Titles	320 (25.8)	922 (74.2)	1242 (100)	1
Abstract sentences	775 (7.5)	9535 (92.5)	10,310 (100)	0.991
Link sentences	441 (34.3)	845 (65.7)	1286 (100)	0.988

Table 2 shows some sample sentences extracted from the training set. The columns show the articles’ annotation, while the rows indicate the annotation per sentence (ie, titles and abstract sentences). A positively annotated sentence implies that the manuscript contains a database. Conversely, negatively annotated sentences can be found in positive and negative articles. Specifically, the sentence “Mobility changes in response to COVID-19” is the title of an article that presents a free access database to the community [15]; however, the title does not show evidence of this and, therefore, receives a negative label. Most of the abstract sentences do not present direct evidence of mentioning a database. On average, we found that only 2 sentences from the abstracts of papers that confirmed to be reporting a database were also positively annotated.

**Table 2:** Examples of trained sentences. Annotation of title and abstract sentences extracted from annotated articles.

Category and annotation of the trained set	Positive article (database found)	Negative article (database not found)
Positive title	CoV2ID: Detection and therapeutics oligo database for SARS-CoV-2	N/A^a^
Negative title	Mobility changes in response to COVID-19	Predicting care and repercussions for caregivers of surgical patients at home
Positive abstract sentence	We have created a comprehensive manually curated database of circular RNAs associated with diseases.	N/A
Negative abstract sentence	Recent studies have shown the role of circRNAs in a number of diseases.	A non-randomized and consecutive sample of 317 informal caregivers of surgical patients with abdominal surgery was included in the study.

^a^N/A: not applicable. No positive sentence from this category could be found in articles where no database was found.

## Results

### Overview

**Figure 6:**
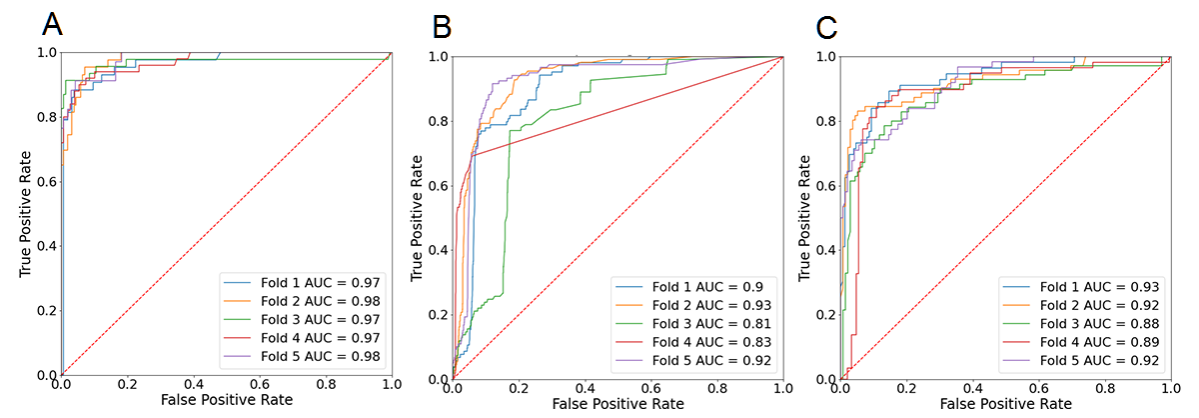
Receiver operating characteristic curve and area under the curve (AUC) values for the 5-fold cross-validation experiment on the three trained models. The dotted diagonal lines represent random chance. (A) Title classification; (B) abstract classification; (C) link classification.

In Figure 6, we present the performance of the three models in their particular tasks. We assessed the models with a 5-fold cross-validation approach. We show the receiver operating characteristic curves and the associated area under the curve (AUC) values in the figure.

As can be seen, the title classification model obtained very high AUC values for every fold, all of them above 0.95, showing high precision and recall scores under the different data partitions. Conversely, the model for abstract sentences achieved high AUC values (above 0.90) on some of the divisions but not on all of them, as two of them presented a score ranging between 0.80 and 0.85. Finally, the link sentences model showed consistent performance, with every AUC score within a 0.03 distance from 0.90.

We also evaluated the whole system by applying the ensemble model on database publication detection, following the algorithm described in Figure 4, and comparing the results to those obtained with its individual components alone, namely the title model and the abstract sentences. This validation was made with a single partition of 30% and 70% for training over the samples.

Note that this task was not the same as the one performed in the previous experiment. We illustrate this with an example: if an article shows evidence of reporting a new database in the abstract but not in the title, such a title is assigned a negative label. In contrast, the paper as a whole is labeled as a positive sample. When we apply the title model to the aforementioned article title, if the model outputs a negative prediction, it is considered a hit in the cross-validation experiment. However, it is a miss for the ensemble model evaluation experiment since the predicted label does not match the expected class.

In Table 3, we present the results regarding precision, recall, and F1 score. The highest precision was obtained with the title model, but this model also yielded the lowest recall score. On the other hand, the abstract sentences model had a lower precision but achieved a better F1 score. Finally, we can see from the table that the ensemble model yielded the best recall and the best F1 score, showing a higher overall performance than both individual models.

**Table 3:** Precision, recall, and F1 scores for the title sentence model, the abstract sentences model, the ensemble model on the article classification task, and the weblinks.

Category	Precision	Recall	F1 score
Title model	0.960	0.795	0.870
Abstract model	0.911	0.923	0.917
Ensemble (title + abstract)	0.900	0.959	0.929
Weblinks	0.922	0.893	0.908

To build the BiDI database, we applied the ensemble model to all the articles collected from PubMed, PMC, and the COVID-19 Open Research Dataset (CORD-19). The total number of manuscripts that received a positive annotation, after removing duplicates, was 10,417: 5354 from PubMed, 5001 from PMC, and 62 from CORD-19. It is important to note that we found very few false positive cases in this list. One example was the article with the PubMed identifier 32226598, which was positive due to some sentences having reported computer resources and because it included an “Associated Data” category on the article’s header, but it was empty (ie, “Not applicable”).

### Use Cases

BiDI provides a search engine based on the Medical Subject Headings (MeSH) vocabulary [16]. This taxonomy, included in the UMLS Metathesaurus, is a controlled vocabulary, a collection of medical-related terms. The NLM uses MeSH terms to classify PubMed articles. As of January 2020, the taxonomy contains more than 29,000 elements. By allowing the user to filter papers by these terms, we enable the application of very specialized queries.

**Figure 7:**
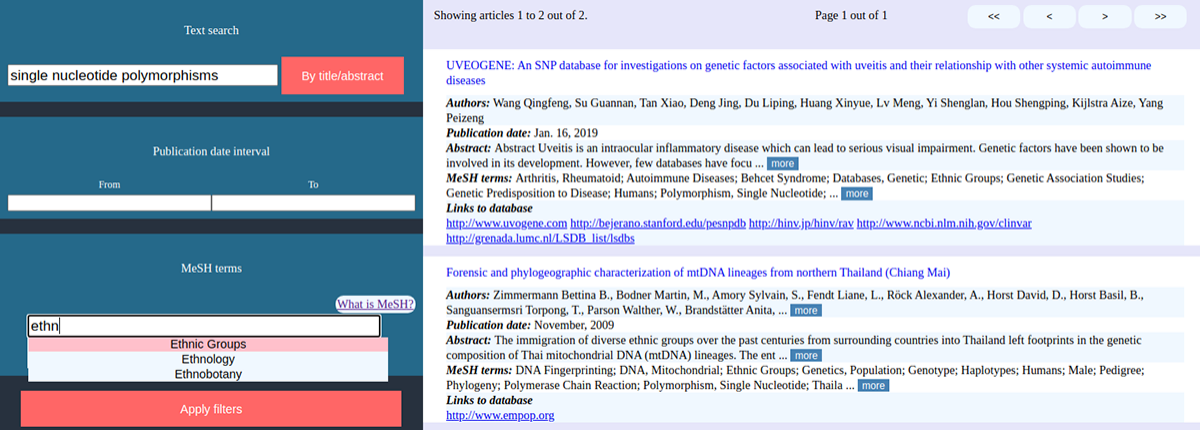
Search results for single nucleotide polymorphisms databases in the Biomedical Database Inventory (BiDI) web platform.

To demonstrate the utility provided by BiDI, we now present two use cases. Let us suppose that we want to find and access data repositories on single nucleotide polymorphisms for specific ethnic populations. Through BiDI, we performed a text search by typing “single nucleotide polymorphism,” accepting matches from either the title or the abstract. BiDI returned a total of 230 articles with associated databases. We then applied MeSH term filters to narrow the search. We selected the “Ethnic Groups” MeSH term, and after proceeding with the query expansion, BiDI presented two papers reporting databases relevant to our query. The final results are shown in Figure 7.

Similarly, suppose that we want to find genomic databases related to coronavirus. We performed a text search by typing “coronavirus.” A total of 16 results were returned, including repositories with heterogeneous data types, such as fatality rates or Twitter messages regarding coronavirus. To narrow the search, we selected the “Genomics” MeSH term. The query returned three articles reporting coronavirus genomic databases.

## Discussion

The number of publications and associated databases included in BiDI is one order of magnitude higher than those of manually collected database repositories. The possibility to perform automatic and regular updates is also a significant advantage. In that sense, our NLP system can analyze up to 42 articles per second on an average commercial graphics card, therefore updating thousands of manuscripts in minutes instead of hours or days, as would be expected if a team of human curators did the work.

Concerning general-purpose repositories such as PubMed, we consider BiDI a complement to them. Moreover, a PubMed weblink is provided along with every article when clicking on the title. BiDI cannot be directly compared to PubMed, as the former is a specialized subset of the latter, and it is not intended to be a replacement but an extension of PubMed. BiDI’s objective is to provide fast access to databases and their associated articles; therefore, a link to the data is included along with every paper. The PubMed platform does not directly acknowledge the resources presented in articles since its goal is to provide generic access to a large number of manuscripts.

Regarding performance, BiDI has shown high precision and recall scores with a training data set of moderate size. We can only expect better performance and generalization with more training data given the superior data scalability provided by deep learning models.

Given that we only considered articles providing URLs, the resulting repository acts as a direct link between the papers and the actual data sets; it then essentially becomes a bridge between research and data, as proposed in earlier studies. In particular, Hoogerwerf et al [17] described the efforts made by the OpenAIRE initiative [18] to promote discipline-independent linking practices between publications, data, project information, and researchers. BiDI complies with these guidelines and, in the future, it could be expanded to integrate project metadata and author information.

BiDI’s mission is to make scientific database resources easier to find and easy to access to facilitate biomedical scientists’ and clinicians’ routine work. Many authors currently do not provide easy access to their experimental data, after a deidentification process to prevent personal data rights violations. In this context, many initiatives have been launched to increase data sharing in science. For instance, the Findable, Accessible, Interoperable, and Reusable (FAIR) Principles [19] defined a set of recommendations focused on improving findability, accessibility, interoperability, and scientific data reusability. BiDI contributes mainly to the first two principles and aims to extend its service to the community as more researchers align themselves with the FAIR Principles and share their data to catalyze scientific discovery.

We may think of medical imaging and the “omics” fields as obvious candidates to benefit from BiDI, due to the amount of data generated by experiments in those areas. However, almost any domain can benefit from our repository to a certain extent. Others can easily reimplement the approach to create their own search tools. Ultimately, BiDI may enable the reuse of biomedical resources and facilitate data-driven research and other scientific initiatives.

We integrated BiDI into an automatic text processing pipeline to update the repository regularly. BiDI is openly available online [20].

## Abbreviations

AUC: area under the curve
BiDI: Biomedical Database Inventory
[CLS]: classification token
CORD-19: COVID-19 Open Research Dataset
DoD2007: Database of Databases
FAIR: Findable, Accessible, Interoperable, and Reusable
MeSH: Medical Subject Headings
NAR: Nucleic Acids Research
NLM: National Library of Medicine
NLP: natural language processing
OMIM: Online Mendelian Inheritance in Man
PMC: PubMed Central
SNOMED: Systematized Nomenclature of Medicine
UMLS: Unified Medical Language System
